# The Role of Cytochrome P450 AbyV in the Final Stages of Abyssomicin C Biosynthesis

**DOI:** 10.1002/ange.202213053

**Published:** 2022-12-08

**Authors:** Andrew J. Devine, Alice E. Parnell, Catherine R. Back, Nicholas R. Lees, Samuel T. Johns, Ainul Z. Zulkepli, Rob Barringer, Katja Zorn, James E. M. Stach, Matthew P. Crump, Martin A. Hayes, Marc W. van der Kamp, Paul R. Race, Christine L. Willis

**Affiliations:** ^1^ School of Chemistry University of Bristol BS81TS Bristol UK; ^2^ School of Biochemistry University of Bristol BS81TD Bristol UK; ^3^ School of Natural and Environmental Sciences Newcastle University NE17RU Newcastle-upon-Tyne UK; ^4^ BioPharmaceuticals R&D AstraZeneca Pepparedsleden 1 43183 Mölndal Sweden

**Keywords:** Antibiotics, Biosynthesis, P450 Enzymes, Polyketides, Structure Elucidation

## Abstract

Abyssomicin C and its atropisomer are potent inhibitors of bacterial folate metabolism. They possess complex polycyclic structures, and their biosynthesis has been shown to involve several unusual enzymatic transformations. Using a combination of synthesis and in vitro assays we reveal that AbyV, a cytochrome P450 enzyme from the *aby* gene cluster, catalyses a key late‐stage epoxidation required for the installation of the characteristic ether‐bridged core of abyssomicin C. The X‐ray crystal structure of AbyV has been determined, which in combination with molecular dynamics simulations provides a structural framework for our functional data. This work demonstrates the power of combining selective carbon‐13 labelling with NMR spectroscopy as a sensitive tool to interrogate enzyme‐catalysed reactions in vitro with no need for purification.

## Graphical abstract

The cytochrome P450 enzyme AbyV catalyses a key epoxidation in the final stages of the biosynthesis of the spirotetronate antibiotic abyssomicin C. Combining structural and computational data with a ^13^C labelling strategy was found to be a powerful approach to interrogate the biotransformation and determine the precise function of the enzyme.

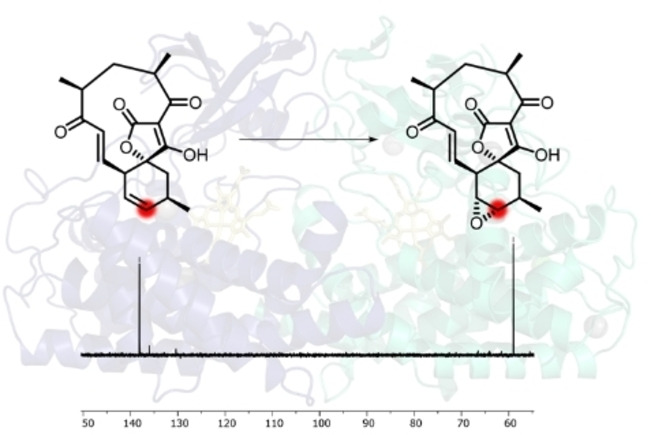

## Introduction

**Scheme 1:**
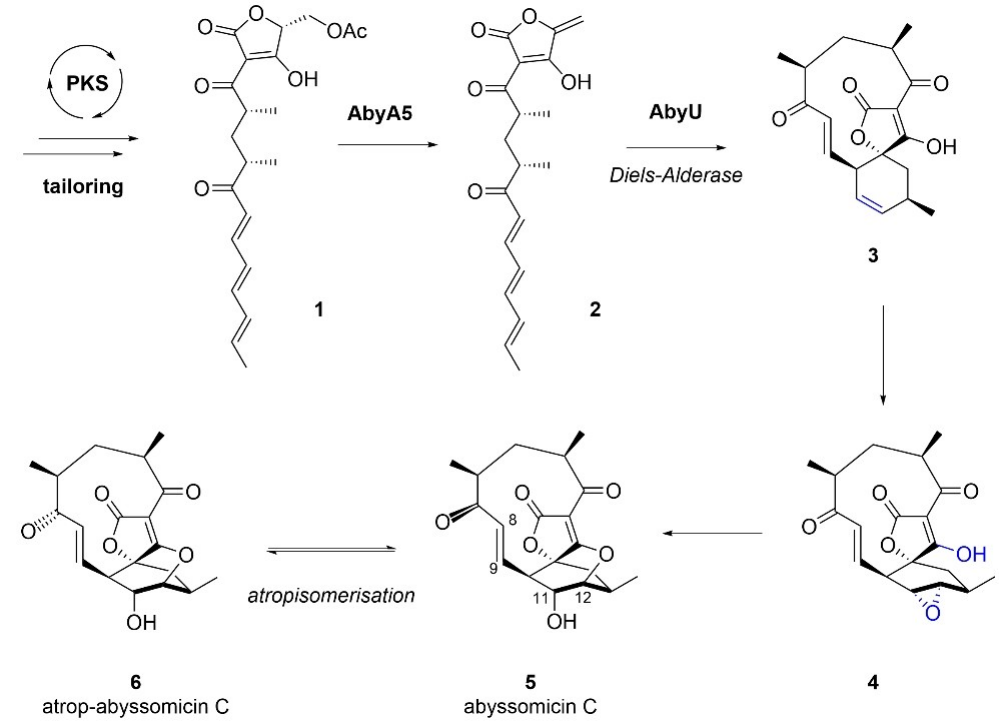
Late stages of abyssomicin C biosynthesis in *M. maris*.

Spirotetronates and spirotetramates comprise a family of polyketide natural products characterised by a macrocyclic structure containing a tetronic acid or tetramic acid moiety fused to another ring system via a spiroatom.^[1]^ These compounds are of widespread interest due to their complex architectures, potent antimicrobial and/or antitumour activities, and their biosynthetic pathways, which incorporate many unusual enzymatic steps. Abyssomicin C is a spirotetronate antibiotic produced by the marine bacterium *Micromonospora maris* (originally *Verrucosispora maris*, reclassified in 2018^[2]^). It is a potent inhibitor of bacterial folate metabolism, showing activity against a variety of clinically relevant bacterial pathogens including *Mycobacterium tuberculosis* and multi‐drug resistant strains of *Staphylococcus aureus*.[^3^, ^4^] In 2010 Süssmuth and co‐workers identified the gene cluster (*aby*) responsible for abyssomicin C production, comprising a multi‐modular polyketide synthase and associated tailoring and regulatory proteins.^[5]^ Following investigations involving feeding studies, comparative bioinformatic analyses, and in vitro experiments, a biosynthetic pathway to abyssomicin C was proposed (Figure S1). Key steps include formation of tetronate **2** via a stereospecific acetate elimination, catalysed by AbyA5,^[6]^ and a subsequent Diels–Alderase (AbyU) promoted intramolecular [4+2]‐cycloaddition which generates **3** with the spirocyclic framework of abyssomicin C (Scheme 1).^[7]^

A closely related conformational isomer of abyssomicin C **5**, atrop‐abyssomicin C **6**, has also been isolated from culture extracts of *Micromonospora maris*.^[8]^ The presence of the two distinct abyssomicin C isomers was first noted in Sorensen's 2005 total synthesis,^[9]^ however, it was not until Nicolaou's 2007 total synthesis that the structural differences between the two species were characterised.^[10]^ The two isomers can be interconverted in the presence of acid and their respective X‐ray crystal structures revealed subtle structural deviations of the C_11_ macrocycle, resulting in the C‐7 carbonyl adopting a transoid relationship with the 8,9‐double bond in abyssomicin C and a cisoid relationship in atrop‐abyssomicin C (Scheme 1). The two compounds target bacterial *p*ABA (*p*‐aminobenzoic acid) biosynthesis by inhibiting the enzyme aminodeoxychorismate synthase, with the oxabicyclo[2.2.2]octane core closely resembling a solution state conformation of the enzyme's substrate chorismate.^[11]^ The ether bridge has been proposed to originate via a biosynthetic epoxidation of the cyclohexene ring of **3** to give **4**, followed by regioselective attack of the tetronate enol to give the natural product (Scheme 1). Three candidate oxygenases for the proposed epoxidation step were identified by Süssmuth (AbyE, AbyV and AbyX).^[5]^ HPLC‐MS chemotyping of the respective *M. maris* knockout mutants failed to detect any predicted biosynthetic intermediates, however only in the strain in which the cytochrome P450 gene *abyV* had been inactivated was abyssomicin C production completely abolished.

The ability to oxidise complex scaffolds with remarkable selectivity has made cytochrome P450 enzymes attractive targets for practical applications.^[12]^ A thorough understanding of the roles and mechanisms of P450s involved in polyketide biosynthesis will undoubtedly enhance and expand our ability to diversify polyketide scaffolds through pathway engineering and biocatalytic approaches. Using a combination of synthesis, isotopic labelling, in vitro assays, X‐ray crystallography and computational simulations, we now reveal the role of the P450 enzyme AbyV in abyssomicin C biosynthesis.

## Results and Discussion

The gene encoding AbyV was amplified from *M. maris* genomic DNA and inserted into the plasmid pOPINF.^[13]^ This construct (pOPINF::abyV) was used to facilitate the recombinant over‐expression of N‐terminally hexa‐histidine tagged AbyV in *E. coli* BL21(DE3) cells. The resulting protein was purified to homogeneity (Figure S2), giving an orange‐brown enzyme solution that was characterised by UV/Visible spectroscopy. The purified protein showed an absorbance maximum at 420 nm, which upon reduction with sodium dithionite shifted to 410 nm concomitant with a modest reduction in peak intensity. Purging the dithionite reduced solution with CO gave the characteristic Fe^II^‐CO complex absorbance spectrum with a maximum at 450 nm, thus providing physical evidence that AbyV is a cytochrome P450 (Figure S3).

To probe the function of AbyV we opted to employ an in vitro reconstitution approach analogous to that used previously by others in the study of P450 enzymes.[^14^, ^15^] A sample of the proposed cyclohexene substrate **3** was synthesised by adapting the approach described in our earlier studies of the Diels–Alderase, AbyU (Figure S4).^[7]^ Titration of this substrate into a buffered solution of AbyV enabled determination of the substrate dissociation constant (*K*_d_) of **3** for AbyV, which was found to be 34±7 μM (Supporting Information and Figure S5). Bacterial P450 catalysis relies on electrons from NAD(P)H co‐factors, shuttled to the heme centre by reductase and ferredoxin reductase partner proteins. However, as the native ferredoxin had not been identified in the *aby* biosynthetic gene cluster (BGC), the widely used heterologous spinach reductase system (FdR/FdX) was selected to reconstitute AbyV activity in vitro.[^16^, ^17^]

**Figure 1:**
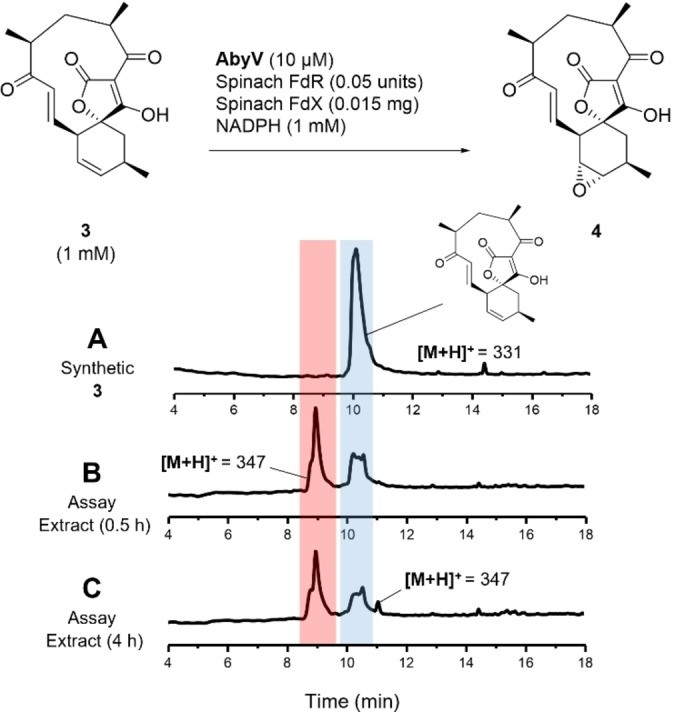
AbyV functional assay using the spinach reductase system. Reverse‐phase LC–MS UV traces (5–95 % MeCN in H_2_O, 0.05 % formic acid) showing A) Synthetic standard of **3**. B) Assay extract following incubation of **3** with AbyV assay mixture for 0.5 h. C) Assay extract following incubation of **3** with AbyV assay mixture for 4 h. Peak broadening is likely to be due to tautomers of the tetronic acids.

Spirotetronate **3** was incubated for 0.5 hours at 25 °C with recombinant AbyV, spinach ferredoxin‐NADP^+^ reductase, spinach ferredoxin and NADPH. LC–MS analysis of the organic extracts revealed the partial conversion of the substrate to a new more polar species (*t*_R_=9.0) with a mass ([*M*+H]^+^=347) consistent with the expected addition of one oxygen atom (Figure 1B). In control assays lacking AbyV only the starting material **3** was observed by LC–MS. To investigate if the biotransformation could be taken to completion, time course experiments (1 h, 2 h and 4 h) were undertaken. After 4 hours, LC–MS revealed conversion of the substrate **3** to the previously observed more polar species (*t*_R_=9.0), but with no apparent change in the ratio of substrate to product (Figure 1C). To investigate if this apparent stalling of the biotransformation was due to one of the assay components being a limiting factor, the reactions were repeated, but after 2 hours were supplemented with fresh AbyV and NADPH. However, no change in the ratio of substrate to product was observed under these conditions. Interestingly, the LC–MS trace of the extract from the 4 hour assay revealed the presence of a new minor peak (*t*_R_=11.1), again with the same mass as the initially observed product ([*M*+H]^+^=347). These observations were consistent with an AbyV catalysed oxidation of substrate **3** giving one major product (epoxide **4**), and the retention time of the new peak apparent after 4 hours was in accord with abyssomicin C **5** or atrop‐abyssomicin C **6**.

To isolate a sample of the major product (*t*_R_=9.0) the mixture was purified by reverse‐phase HPLC (5–95 % MeCN in H_2_O, 0.05 % formic acid). ^1^H NMR analysis of the purified product (Figure S6) confirmed the presence of epoxide **4** with characteristic signals at δ 3.03 and δ 3.17 assigned to 12‐H and 11‐H respectively. Interestingly, atrop‐abyssomicin C **6** was also evident as a minor component in the sample. This observation was consistent with the oxirane ring in **4** having undergone in situ intramolecular ring opening by attack from the OH of the tetronic acid to form the ether bridge, presumably promoted by formic acid from the HPLC solvent system. This apparent sensitivity to the acidic HPLC solvent would also account for the appearance of the minor product in the 4 hour assay extract (Figure 1C), arising from the same in situ epoxide opening of the initial assay product.

**Scheme 2:**
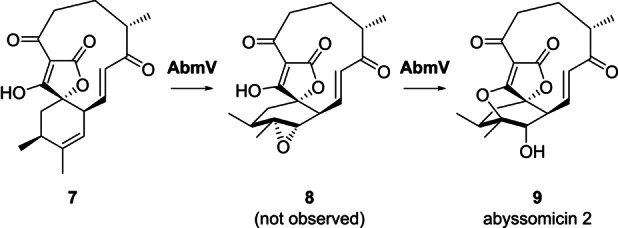
Final steps of abyssomicin 2 biosynthesis in *S. koyangensis* proposed by Ju following in vitro studies with AbmV.^[18]^

The LC–MS and NMR data were in accord with the proposal that AbyV catalyses oxidation of alkene **3** to epoxide **4** in abyssomicin C biosynthesis, but not the final step: intramolecular attack on the epoxide to form the ether bridge. These observations differ from the conclusions drawn by Ju and co‐workers working with the cytochrome P450 enzyme AbmV from the closely related abyssomicin 2 biosynthetic pathway in *Streptomyces koyangensis*.^[18]^ They conducted in vitro assays with recombinant AbmV, substrate **7** and reductase partner proteins from *Synechococcus elongatus* PCC 7942 (Scheme 2). LC–MS analysis of the assay extracts revealed a new peak with a significant shoulder and the mass observed for this product peak was 347.1495 ([*M*+H]^+^). The authors concluded that the product was abyssomicin 2 (**9**) and that broadening of the signal might be due to a tautomer of **9**. Hence, it was proposed that AbmV is responsible for catalysing both epoxidation and subsequent etherification via epoxide opening (Scheme 2). A significant structural difference in the natural substrates for AbmV and AbyV (**7** and **3** respectively) is that **7** has a trisubstituted alkene in the 6‐membered ring whereas **3** has the disubstituted 11,12‐alkene. The structural differences in the resultant epoxides (**8** from AbmV and **4** from AbyV) could then influence the rate of attack to form the cyclic ethers (**9** and **5**/**6**).

In our studies of abyssomicin C biosynthesis, we were confident that epoxide **4** was the major product from the AbyV assay (Figure 1). However, the presence of trace amounts of atrop‐abyssomicin C in the ^1^H NMR spectrum of the purified assay product and in the 4 hour assay extract led to some uncertainty as to the role of AbyV in the intramolecular epoxide opening step, even though it clearly occurred in the absence of enzyme on exposure to formic acid present in the HPLC solvents. Hence the extent to which atrop‐abyssomicin C formation was promoted by acid or enzyme catalysis needed to be investigated further.

**Scheme 3:**
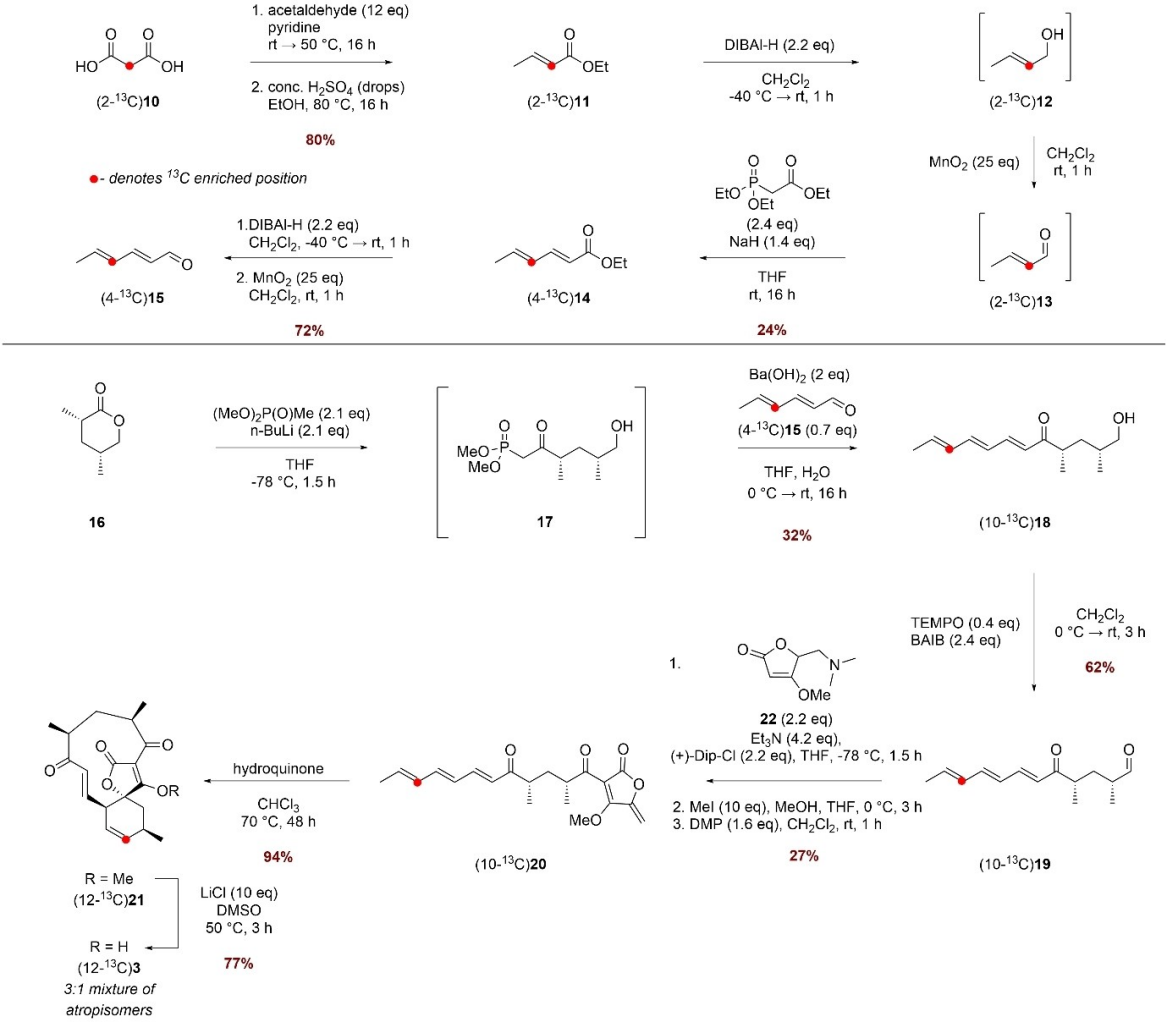
Synthesis of (12‐^13^C)**3**.

Thus, we turned our attention to a selective carbon‐13 labelling based NMR assay to monitor the catalytic activity of AbyV since this would obviate the need for purification of the product mixture prior to analysis. The ideal site for incorporation of the isotopic label was C‐12 as the chemical shifts of the signals in the ^13^C NMR of the starting alkene **3** and likely products (epoxide **4** and the atropisomers of abyssomicin C **5** and **6**) would be well‐resolved (CD_3_OD δ 137.3, 58.9, 85.1 and 86.8 respectively).[^8^, ^10^] (12‐^13^C)Alkene **3** was prepared as shown in Scheme 3 using commercially available (2‐^13^C)malonic acid **10** as the source of isotopic label. Knoevenagel–Doebner condensation of (2‐^13^C)malonic acid **10** and acetaldehyde followed by esterification gave ethyl (2‐^13^C)crotonate **11** in 80 % yield over the 2 steps. The subsequent steps were conducted without purification of the intermediates due to the challenge of working with volatile materials. Thus, sequential reduction of **11** to alcohol **12** using DIBAL‐H followed by oxidation with MnO_2_ gave (2‐^13^C)crotonal **13** which was used directly in a Horner–Wadsworth–Emmons (HWE) olefination with triethyl phosphonoacetate to give unsaturated ester (4‐^13^C)**14** in 24 % yield over the three steps. A similar two‐step reduction/oxidation protocol converted ester **14** to (4‐^13^C)aldehyde **15** in 72 % yield. Freshly prepared (4‐^13^C)**15** was then chain extended via a HWE olefination with phosphonate **17** giving triene (10‐^13^C)**18** in 32 % yield from lactone **16**. (10‐^13^C)**18** was then oxidised to the corresponding aldehyde, which was reacted with tetronate **22** in a Dip‐Cl promoted vinylogous aldol reaction. Treatment with methyl iodide and subsequent oxidation with DMP gave linear ketone (10‐^13^C)**20** with the *exo*‐methylene tetronate ring installed. A biomimetic thermal Diels–Alder reaction converted the linear compound (10‐^13^C)**20** to spirotetronate (12‐^13^C)**21** and finally demethylation with LiCl/DMSO gave (12‐^13^C)**3**, the required substrate for the in vitro assays with AbyV.

**Figure 2:**
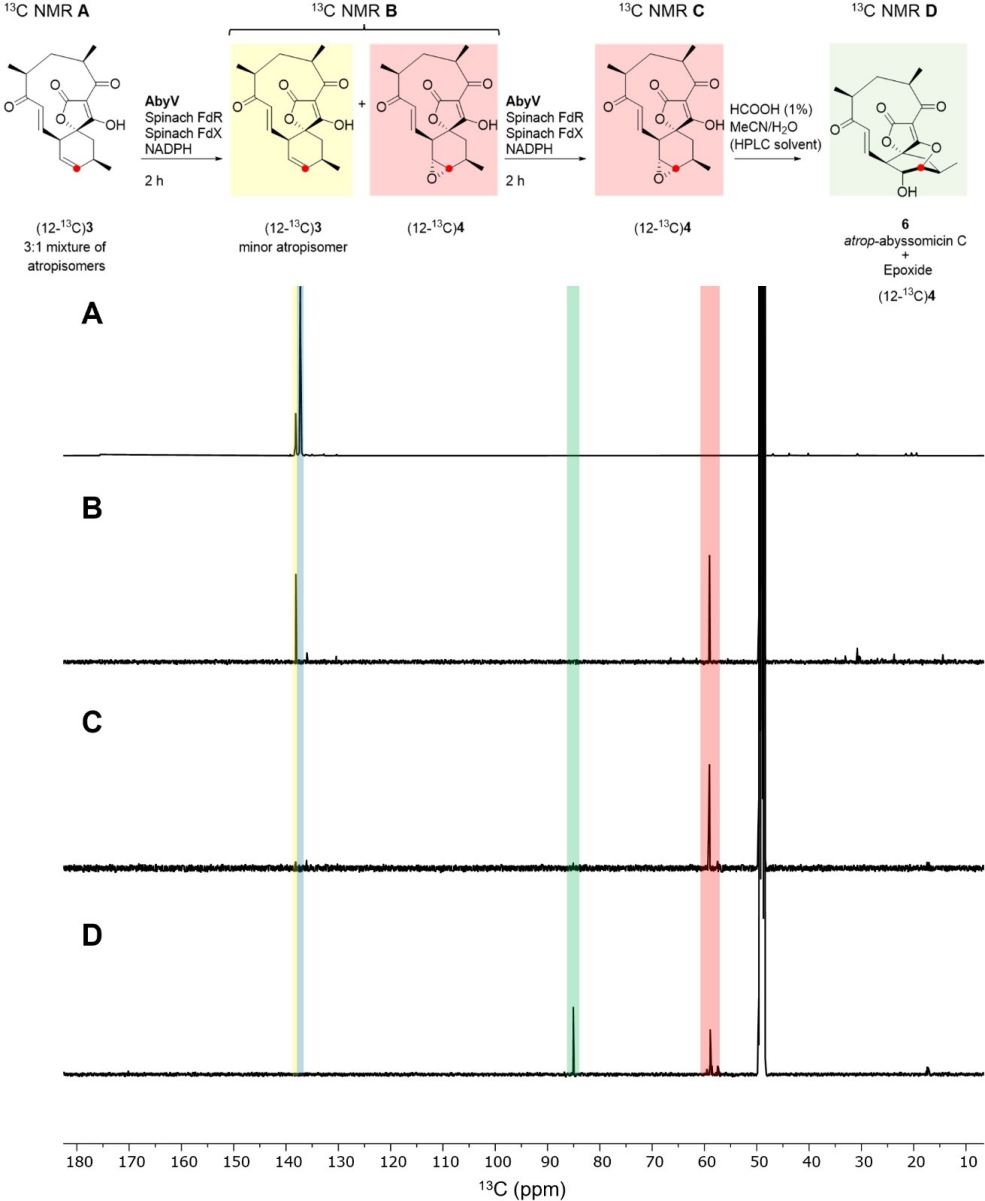
^13^C NMR Spectra (125 MHz, CD_3_OD): A) Substrate (12‐^13^C)**3** as a mixture of atropisomers B) Crude extract following incubation of (12‐^13^C)**3** with AbyV, spinach ferredoxin‐NADP+ reductase, spinach ferredoxin and NADPH for 2 hours showing formation of epoxide (12‐^13^C)**3**. C) The assay mixture from (B) was dried, then the in vitro assay was repeated giving solely (12‐^13^C)epoxide **4**. D) (12‐^13^C)Epoxide **4** from (C) was purified by RP‐HPLC giving a mixture of unchanged epoxide (12‐^13^C)**4** and atrop‐abyssomicin C **6**.

The ^1^H NMR spectrum of methyl tetronate (12‐^13^C)**21** was in accord with that of unlabelled **21**, with characteristic signals assigned to the enone (9‐H: CDCl_3_ δ 6.47 (1*H*, dd, *J* 17.0, 7.0) and 8‐H δ 6.97 (1*H*, d, *J* 17.0)). These signals closely align to the corresponding signals for atrop‐abyssomicin C indicating that the enone in both compounds adopts the same conformation. As expected, the main difference compared with the spectrum of unlabelled material was the signal assigned to 12‐H which showed characteristic ^1^H‐^13^C coupling: δ 5.85 (1H, app. ddt, *J* 149.5, 10.0, 3.0). The enhanced signal at δ 136.7 assigned to C‐12 in the ^13^C NMR spectrum of (12‐^13^C)**21** was accompanied by a minor slightly downfield signal in a ratio of approximately 5 : 1, as observed in previous syntheses.^[7]^ Following demethylation, the signals in the ^1^H NMR spectrum of (12‐^13^C)**3** were broad due to tautomerism of the free tetronic acid. The ^13^C NMR spectrum revealed a 3 : 1 mixture of major to minor isomers, clearly visible in the enhanced C‐12 signals shown in Figure 2A. LC–MS analysis showed the presence of a single peak with the expected mass, and the two species were chromatographically inseparable. The presence of two enriched signals in the ^13^C NMR spectrum was attributed to the presence of atropisomers, as reported for the natural product, and this material was used in in vitro AbyV assays.

First, assays were conducted by incubation of (12‐^13^C)**3** with AbyV, spinach ferredoxin‐NADP+ reductase, spinach ferredoxin and NADPH. After two hours the assay mixture was extracted with ethyl acetate and the crude mixture analysed by ^13^C NMR (Figure 2B). Two signals were clearly visible, one at δ 138.1 ppm (assigned to the enhanced signal of the minor atropisomer of substrate **3**) and the other at δ 59.0 ppm, in accord with the chemical shift of the C‐12 position of a synthetic sample of epoxide **4** (for synthetic details see Supporting Information Figure S4). No trace of abyssomicin C or atrop‐abyssomicin C was detected in the ^13^C NMR spectrum of the crude mixture (expected signals for C‐12, 85.1 and 86.8 ppm).[^8^, ^10^] These data are in accord with the LC–MS assay using unlabelled substrate **3** in which the reaction did not reach completion and with epoxide **4** as the major product (Figure 1). The assay was then conducted in an NMR tube to enable in situ monitoring of the reaction by ^13^C NMR. Partial conversion to epoxide **4** was again observed and no cyclic ether, **5** or **6**, was detected (Figure S7). To investigate if the AbyV catalysed reaction could be taken to completion, the crude mixture from the initial assay containing both unreacted substrate (12‐^13^C)**3** and epoxide **4** (Figure 2B) was dried and then resuspended in a fresh AbyV assay mixture for two hours. In this case full conversion of the substrate was observed giving epoxide **4** as the only product evident by ^13^C NMR (Figure 2C).

Finally, to verify the role of the acidic conditions during HPLC purification in the formation of atrop‐abyssomicin C from epoxide **4**, the sample of (12‐^13^C)epoxide **4** obtained from the dual enzyme assays (Figure 2C) was purified by HPLC and the product analysed by ^13^C NMR (Figure 2D). As expected, two signals were observed, one corresponding to epoxide **4** and one new downfield signal at 85.1 ppm, which is consistent with the chemical shift of C‐12 in atrop‐abyssomicin C **6**.^[8]^ This confirms that epoxide **4** is readily converted to the natural product upon exposure to acid.

**Figure 3:**
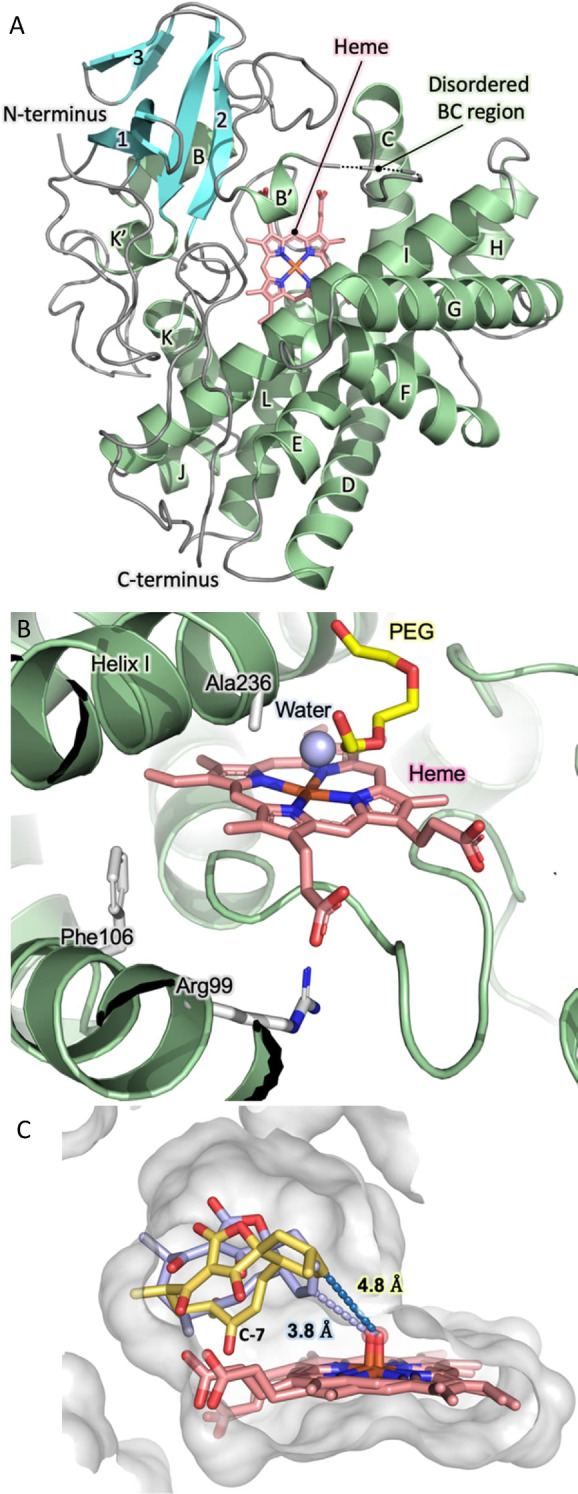
Crystal structure of AbyV and mode of substrate binding. A) Overall fold of AbyV highlighting individual secondary structure elements, the disordered BC region and the active site bound heme cofactor. B) AbyV enzyme active site C) Representative orientations from simulation of the binding modes of the major (yellow) and minor (blue) atropisomer of **3** in the AbyV active site. Heme (as Compound I) is also shown.

All the results described above were reproducible and fully consistent in providing unambiguous evidence to support the proposal that AbyV catalyses solely epoxidation of **3** to give **4** in abyssomicin C/atrop‐abyssomicin C biosynthesis. Next, we sought to provide a structural framework for the AbyV catalysed reaction. Purified recombinant AbyV was subjected to crystallisation screening, with resulting crystals submitted for X‐ray diffraction analysis using synchrotron radiation. The crystal structure of AbyV (PDB ID 7QAN, Figures 3A and B) was determined to a 2.01 Å resolution by molecular replacement using Chain A of the *Streptomyces avermitilis* P450CYP105D6 (PDB ID 3ABB, 50 % sequence identity with AbyV) as the search model (Table S1).^[17]^ The crystal structure of AbyV reveals an asymmetric unit comprising two molecules of the polypeptide (Chains A and B), with Chain A accounting for residues 11–395 of the full‐length protein and Chain B residues 27–395. Electron density was not observed for residues 81–87 of Chain A and 73–94 of Chain B, consistent with the assignment of this region as the highly flexible BC loop. The overall fold of AbyV is typical of a cytochrome P450^[19]^ with a large α‐helical domain (helices B–L) and a smaller β‐sheet region (sheets 1–3). Surprisingly, the region corresponding to helix “A” in other P450s forms a loop in AbyV, hence our assignment of the first helix of AbyV as “B”. This peculiarity was further investigated by modelling the structure of AbyV using AlphaFold2 (ColabFold implementation)[^20^, ^21^, ^22^] with the resulting model being fully consistent with our crystal structure and indicating that the N‐terminal residues are indeed disordered (pLDDT score for 1–8 <50, 58 for residue 9, 77 for residue 10; only sufficient electron density from residue 11 onwards); and that residues 11–32 form a loop structure without any alpha‐helical content.

Each molecule of AbyV contains a single heme group situated within a large solvent exposed active site pocket of ≈1600 Å^3^, as calculated using CASTp.^[23]^ Recalculation of this value using a homology model of the polypeptide with a fully structured BC loop yielded an equivalent active site volume. The heme cofactor of AbyV is coordinated by the side chain of residue Cys344, with a single water molecule observed on the opposing face of the cofactor, 2.1 Å above the heme iron (Figure 3B). Each of the two molecules of AbyV that comprise the AU contain a single molecule of PEG bound within the enzyme active site. Analysis of the AbyV structure using DALI identified a number of closely related P450 enzymes (Supporting Information Figure S8).^[24]^ These include CYP105D18 from *Streptomyces laurentii*, the multifunctional P450 monooxygenase GfsF from *Streptomyces graminofaciens* and the filipin tailoring hydroxylase CYP105D6 from *Streptomyces avermitilis*. Mapping explicitly conserved residues from the ten most structurally related AbyV homologues reveals that the majority of these residues are located in either the conserved heme coordinating loop, common to each of the identified polypeptides, or within tight turns required to achieve appropriate placement of secondary structure elements in the overall protein fold (Supporting Information Figure S9). Our crystal structure provides no evidence for the presence of atypical catalytic features in AbyV such as those reported in other P450 enzymes.^[25]^

To explore the likely enzyme‐substrate interactions of both atropisomers of spirocycle **3**, we used molecular docking to obtain initial positions of **3** near the heme group in chain A of the AbyV crystal structure. We then performed multiple short molecular dynamics simulations for both atropisomers with heme modelled in its compound I state (10× 200 ps runs for each, using the Enlighten protocols,^[26]^ details in the Supporting Information, Figure 3C). Based on the final half of the simulations and the reactivity criteria for epoxidation proposed by Lonsdale, Harvey and Mulholland (Compound I oxygen to epoxidised C distance <4 Å, compound I heme iron, oxygen, epoxidised C angle between 110 and 140°),^[27]^ a higher percentage of reactive poses was observed for one atropisomer of the substrate (81 % vs. 42 %). This is consistent with the atropisomer selectivity observed in the ^13^C labelling experiments (Figure 2), and thus indicates that AbyV‐substrate interactions likely lead to this preference. Our simulations indicate that these interactions are non‐specific, leading to a subtle shift in substrate positioning (with the only notable difference between the atropisomers being that for the non‐preferred atropisomer the C7 carbonyl interacts with the heme propionates; Figure 3C). Notably, the preferred atropisomer is that which results in atrop‐abyssomicin C, which has been indicated as the major product of the biosynthetic pathway and indeed was the only product observed on exposure of epoxide **4** to formic acid.

## Conclusion

In summary, here we report the first structural and functional characterisation of the enzyme AbyV and unambiguously establish its role in abyssomicin C biosynthesis. This cytochrome P450 is responsible for epoxidation of spirocycle **3**, the penultimate step in the biosynthetic pathway and is key to installing the ether bridged core of abyssomicin C, a structural feature critical to its mechanism of action as a substrate mimetic of chorismate. The function of AbyV is distinct from that reported for its homolog AbmV,^[18]^ from the closely related abyssomicin 2 biosynthetic pathway, highlighting the functional versatility of this class of enzyme and the challenges in predicting enzyme function based on sequence similarity. The product of the AbyV catalysed oxidation, epoxide **4**, is unstable under acidic conditions and is converted to the natural product. However, as no epoxide hydrolase has been identified in the abyssomicin BGC, the mechanism by which epoxide **4** is converted to abyssomicin C in the final step of the biosynthetic pathway in *M. maris* remains unknown and is the subject of future studies. Results from ^13^C labelling experiments, in conjunction with structural and computational studies reveal that AbyV is selective for one atropisomer of its natural substrate **3** and therefore may serve as a biosynthetic mechanism for establishing atrop‐selectivity in abyssomicin C biosynthesis. This work further expands our growing understanding of the abyssomicin pathway, and AbyV represents an attractive candidate for incorporation into chemoenzymatic syntheses of abyssomicin C and derivatives thereof.

## Conflict of interest

The authors declare no conflict of interest.

1

## Supporting information

As a service to our authors and readers, this journal provides supporting information supplied by the authors. Such materials are peer reviewed and may be re‐organized for online delivery, but are not copy‐edited or typeset. Technical support issues arising from supporting information (other than missing files) should be addressed to the authors.

Supporting Information

## Data Availability

The data that support the findings of this study are available in the Supporting Information of this article.
